# DisBind: A database of classified functional binding sites in disordered and structured regions of intrinsically disordered proteins

**DOI:** 10.1186/s12859-017-1620-1

**Published:** 2017-04-05

**Authors:** Jia-Feng Yu, Xiang-Hua Dou, Yu-Jie Sha, Chun-Ling Wang, Hong-Bo Wang, Yi-Ting Chen, Feng Zhang, Yaoqi Zhou, Ji-Hua Wang

**Affiliations:** 1 Shandong Provincial Key Laboratory of Biophysics, Institute of Biophysics, Dezhou University, Dezhou, 253023 China; 2 College of Physics and Electronic Information, Dezhou University, Dezhou, 253023 China; 3 Institute for Glycomics and School of Information and Communication Technology, Griffith University, Parklands Dr, Southport, QLD 4222 Australia

**Keywords:** Intrinsic disorder, Database, Function classification, Protein disorder prediction, Protein function, Binding site

## Abstract

**Background:**

Intrinsically unstructured or disordered proteins function via interacting with other molecules. Annotation of these binding sites is the first step for mapping functional impact of genetic variants in coding regions of human and other genomes, considering that a significant portion of eukaryotic genomes code for intrinsically disordered regions in proteins.

**Results:**

DisBind (available at http://biophy.dzu.edu.cn/DisBind) is a collection of experimentally supported binding sites in intrinsically disordered proteins and proteins with both structured and disordered regions. There are a total of 226 IDPs with functional site annotations. These IDPs contain 465 structured regions (ORs) and 428 IDRs according to annotation by DisProt. The database contains a total of 4232 binding residues (from UniProt and PDB structures) in which 2836 residues are in ORs and 1396 in IDRs. These binding sites are classified according to their interacting partners including proteins, RNA, DNA, metal ions and others with 2984, 258, 383, 350, and 262 annotated binding sites, respectively. Each entry contains site-specific annotations (structured regions, intrinsically disordered regions, and functional binding regions) that are experimentally supported according to PDB structures or annotations from UniProt.

**Conclusion:**

The searchable DisBind provides a reliable data resource for functional classification of intrinsically disordered proteins at the residue level.

## Background

More and more proteins are shown to be partially or wholly unstructured or intrinsically disordered [1, 2]. These intrinsically disordered proteins (IDPs) or regions (IDRs) in a protein have a wide variety of functions ranging from molecular recognition, molecular assembly, protein modification to entropic chain activities [3]. Flexible disordered regions offer many unique advantages such as facilitating multiple binding partners, enabling posttranslational modifications and preventing aggregations [4]. Some of IDPs implicated in human diseases are attractive targets for drug discovery [5].

Recognizing the importance of IDPs, several databases have been built. DisProt is the first curated database that contains a collection of experimentally verified IDPs and IDRs [6]. The latest release contains a total of 694 proteins with 1539 disordered regions (a just published newer release expands to more than 800 entries [7] and we will update ours in the next version). D2P2, on the other hand, consists of computationally predicted IDPs from 1765 proteomes from 1256 distinct species [8]. Both computational and experimental annotations were used in MobiDB to annotate >500,000 disordered proteins [9]. Computational annotations relied on a consensus of predictors including IUPRED [10] and ESpritz [11]. Its most recent version [12] further linked to information from post-translational modification in universal protein resource (UniProt) [13] and STRING protein-protein interactions [14]. IDEAL [15] was a database incorporating functional with structural/disorder annotations for 582 IDPs (as of the latest release on 12/Jun/2015) by manually integrating protein data bank (PDB) [16], UniProt [13] and DisProt databases [6]. It has been focused on interaction network of IDPs with induced folding sites annotated in disordered regions.

Here we have compiled a database, DisBind (Disorder Binding sites), which is dedicated to classification of functional binding sites of IDPs and proteins with both intrinsically disordered and structured regions from the DisProt database, regardless if IDPs have or do not have experimentally determined structures by induced folding. Residue-level binding sites are important first step for understanding the functional impacts of genetic variants in coding regions of human and other genomes, considering that a significant portion of eukaryotic genomes code for intrinsically disordered regions in proteins [17]. We categorize binding sites into eight categories according to their binding partners: DNA, RNA, proteins, cofactor/heme, metal ions, substrate/ligand, ATP/GTP, and others. Although some categories only have a few sites, we include them in the database for completeness. This database provides a classification of functional binding sites in IDPs annotated according to experimentally supported evidences. As a comparison, IDEAL does not contain binding sites from metals and ligands. DisProt does not contain binding site information. For completeness, both structured and disordered regions of an intrinsically disordered protein are annotated. Most disordered regions with annotated binding sites do not have known structures. Some disordered regions, however, have experimentally-determined structures when they are in complex with their interaction partners (binding induced folding or conformational selections). For those special cases, we annotated secondary structure motifs involved in binding regions which can provide a basis for initial understanding of binding mechanisms.

## Construction and content

We obtained all annotated IDRs and IDPs from the recent version of DisProt database (v6.02). The binding sites for those IDPs are either retrieved from the annotation of specific binding sites in UniProt and/or derived from the high-resolution complex structures (resolution better than 3.5 Å) in PDB. Most binding sites from UniProt are ion binding sites whereas binding sites from PDB structures are mainly IDP-RNA, IDP-DNA and IDP-protein interactions. For IDPs in a complex structure, binding residues in IDPs are determined by a cutoff distance of 3.5 Å between any atoms of an IDP and its binding partner as with previous studies [18, 19]. Binding partners are classified into 8 categories: DNA, RNA, proteins, cofactor/heme,metal ions, substrate/ligand, ATP/GTP, and others. The secondary structure information of binding residues were also obtained from PDB based on the DSSP (Dictionary of protein secondary structure) assignment [20]. Eight secondary structure groups are combined into three classes i.e. α-helix (H, G, I), β-sheet (B, E) and coil (T, S, D). We note that the link to DSSP only exists for those IDPs with three-dimensional structural regions determined. If the same IDP binds with different proteins associated with different PDB structures, they were annotated separately.

## Utility and Discussion

Current version of DisBind contains 226 IDPs with functional site annotations. These IDPs contain 465 structured regions (ORs) and 428 IDRs according to annotation by DisProt. For completeness, both structured and unstructured regions are annotated. The database contains a total of 4232 binding residues (from UniProt and PDB structures) in which 2836 residues are in ORs and 1396 in IDRs. In Table 1, these binding residues are further classified according to their binding partners. The largest subset of DisBind involves with binding to proteins with 772 binding residues in disordered regions. This followed by 189, 55, and 69 residues in disordered regions that interact with RNA and DNA, and metal ions, respectively. Only a few binding sites are located for the remaining functional categories.

**Table 1:** The number of residues and binding residues of IDPs and IDRs according to binding partners of IDPs in DisBind

Category	# IDPs^a^	# all Residues			# Residues in IDRs		# Binding Residues		
		IDPs^b^	IDRs	ORs	Helix^c^	Sheet^c^	IDPs	IDRs	ORs
ALL	226	166235	29908	136327	1705	439	4232	1396	2836
Protein	127	57586	12822	44764	1299	244	2984	1070	1914
RNA	12	6040	1286	4754	106	131	258	189	69
DNA	32	12092	2853	9239	301	64	383	55	328
Metal	81	40351	6242	34109	-	-	350	69	281
Cofactor	12	6825	1193	5632	-	-	41	2	39
Substrate	32	5791	1014	4777	-	-	61	2	59
ATP/GTP	32	14695	2475	12220	-	-	37	1	36
Others	44	22855	2023	20832	-	-	123	8	115

^a^Some IDPs can bind to different partners. ^b^# of residues or binding residues in IDPs refer to all residues or all binding residues regardless if they are in structured, unstructured, or unannotated regions. ^c^# of helical or sheet residues in IDRs

Please note that IDPs may contain both structured regions and IDRs as well as un-annotated regions

**Fig. 1:**
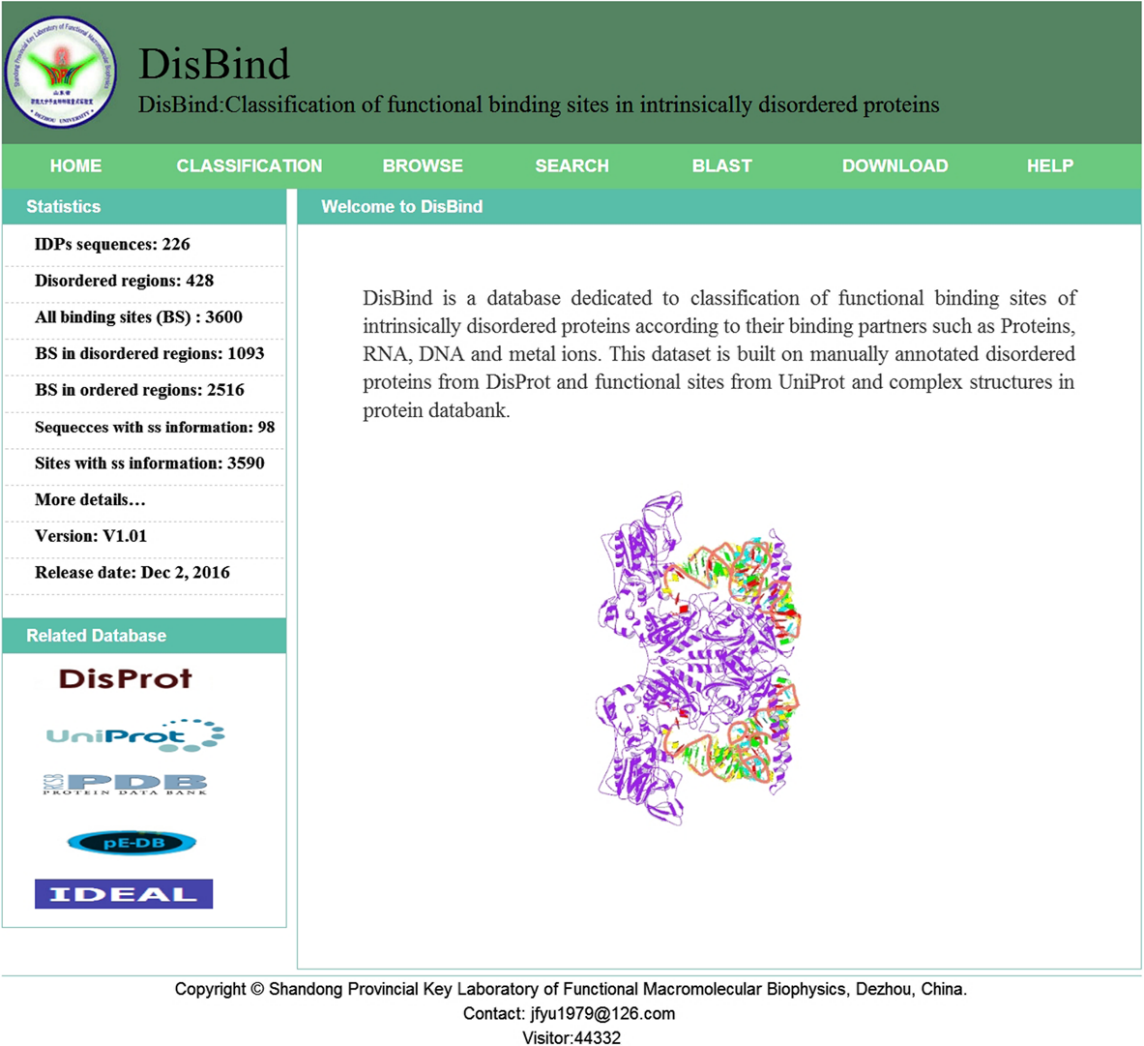
The front page of DisBind database

Figure 1 shows the top page of DisBind which consists of seven parts: ‘Home’, ‘Classification’ , ‘Browse’, ‘Search’, ‘Blast’,‘Download’ and ‘Help’. Under the ‘Classification’ option, the collected items can be retrieved according to their partners (i.e., DNA, RNA, protein, cofactor/heme, metal ions, substrate/ligand, ATP/GTP and others). All items collected in DisBind numbered from N00001 to N00226 can be also retrieved by clicking ‘Browse’ option. Alternatively, a user can obtain the collected information by inputting any keywords by the ‘Search’ option or protein sequences by the ‘Blast’ option. In addition, all of binding sites along with their secondary structures can be downloaded in the fasta format. ‘Help” page contains detailed explanation of each page and meaning of color codes.

**Fig. 2:**
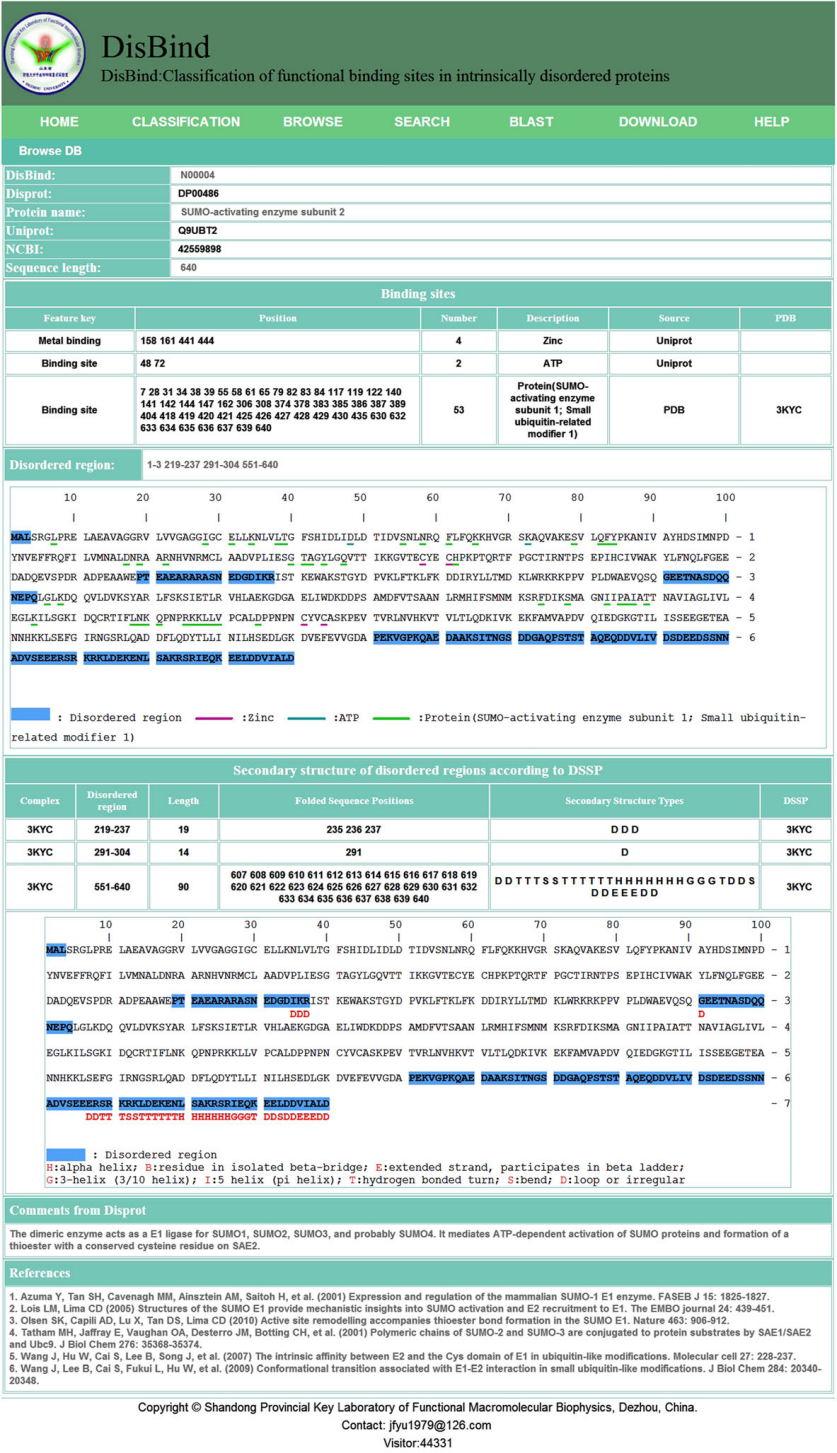
Information collected for each IDP as demonstrated for IDP N00004

The information stored for each IDP has five parts as demonstrated in Fig. 2 by using N00004 as an example. Part I provides the basic information such as identification numbers from DisBind, DisProt, UniProt, and NCBI along with the protein name and its sequence length. Part II contains specific binding sites and corresponding binding partners according to UniProt annotations and/or the PDB complex structure along with PDB ID #. A click on the PDB ID# will directly link to the protein databank for structural visualization. These sites along with annotated disordered regions by DisProt are highlighted in the sequence. The secondary structure in disordered regions is shown along with sequence presented in Part III. Parts IV and V contains comments from DisProt regarding the disordered protein and corresponding references on functional annotations, respectively.

## Conclusion

DisBind is a database dedicated to residue-level classification of functional binding sites in disordered and structured regions of intrinsically disordered proteins. This database compiled information from the structural database (protein databank), the database of experimentally validated disordered proteins (DisProt), and the comprehensive protein sequence and functional database (UniProt). The database is fully searchable and freely accessible. In the next version of the dataset, we will significantly expand the dataset by including disordered proteins (>17000) that are indirectly supported by X-ray crystallography and Nuclear Magnetic resonance collected in MobiDB [12]. Moreover, we plan to incorporate predicted regions using existing techniques such as IUPRED [10] and ESpritz [11] as well as recently accurate developed techniques such as SPOT-Disorder [21]. This large dataset should provide an ultimate resource for functional site classifications in IDPs.

## Availability and requirements

Database homepage: http://biophy.dzu.edu.cn/DisBind. These data are freely available without restrictions for use by academics.

## Abbreviations

DisBind: Database of Disordered protein Binding Sites
DisProt: Database of Protein Disorder
IDPs: Intrinsically Disordered Proteins
NCBI: National Center for Biotechnology Information
PDB: Protein databank
UniProt: Universal Protein Resource
